# Editorial: Food-cravings, body image and eating disorders: perspectives across cultures and genders

**DOI:** 10.3389/fpsyg.2023.1285834

**Published:** 2023-10-04

**Authors:** Antonio Cepeda-Benito, Carla Ugarte Pérez, Paula Lizana-Calderón, Silvia Moreno-Domínguez

**Affiliations:** ^1^Department of Psychological Science, University of Vermont, Burlington, VT, United States; ^2^Departamento de Psicología, Universidad de Jaén, Jaén, Spain; ^3^Centro de Estudios de la Conducta Alimentaria (CECA), Escuela de Psicología, Universidad Adolfo Ibáñez, Santiago, Chile

**Keywords:** food craving, body image, cross-cultural, eating disorders, eating awareness, weight loss

The present Research Topic revives a previous, successful attempt by Frontiers to foster cross-cultural research on phenomena once believed circumscribed to white, upper-middle-class women from the US and other Western, economically rich countries (Swami et al., 2010; Valderrama-Zurián et al., 2017). About 4 years ago, Frontiers published seven original research articles that together studied participants from across five continents, contrasting groups from the same nation but different ethnocultural heritages (Akoury et al., 2019; Wilhelm et al., 2019), or groups from different countries but with a common language and similar cultural heritage (Moreno-Domínguez et al., 2019a), or simply living in vast, highly populous non-Western nations (Chapuis-de-Andrade et al., 2019; Chen et al., 2019). The current, small collection of research articles also represents a wide range of nations and cultures that together contribute to advancing our understanding of eating disorder (ED) phenomena within and outside cultural and geopolitical boundaries.

The idea that body image (BI) dissatisfaction and ED emerge from Western (Anglo) cultural ideals and expectations about women's social roles and bodies grew rapidly from the late 1980's through the 1990's and the first decade of the twenty-first century (Valderrama-Zurián et al., 2017). Although BI and ED research with non-Anglos and people of color began to emerge in the 1990's and grew throughout the early 2000's, interest in BI and ED declined significantly in the second decade of the twenty-first century (Cepeda-Benito and Moreno-Domínguez, 2019). The present Research Topic aimed to examine the connection between food cravings, BI, and ED in different cultural settings. The present Research Topic of articles reports results from four culturally distant countries (Chile, China, Iran, and the US), broaching a broad set of important theoretical, applied, and methodological questions.

Sahlan et al. could not directly compare US and Iranian college students across various ED-related variables because their data lacked metric and scale measurement invariance across samples. These authors settled for conducting separate but parallel analyses to descriptively compare variable correlation patterns across the samples. Sahlan et al. reported that body mass index (BMI), pressure to conform to Western ideals of female beauty, and internalization of those ideals uniquely predicted ED symptoms in both samples (albeit with different degrees of association). Thus, the inclusion of an Iranian sample extended the generalizability of the finding that cultural pressures imposed on women's physical appearance, and that the internalization of these pressures correlate negatively with BI satisfaction (e.g., Warren et al., 2005; Moreno-Domínguez et al., 2019b).

Yan et al. recruited a large, mixed-gendered sample of young adults from six Chinese universities to examine moderated mediational relationships between one antecedent (BI), one mediator (perceived stress), three moderators (self-acceptance, cognitive reappraisal of emotions, and expressive suppression of emotions), and one outcome (binge eating). They found that perceived stress mediated the relationship between BI dissatisfaction and increased binge eating, but that the relationship between BI dissatisfaction and perceived stress was stronger at very low levels of self-acceptance. In addition, although the relationship between perceived stress and binge eating was relatively stronger at low than high levels of emotional regulation, there was no interaction between perceived stress and cognitive reappraisal. Overall, the results were congruent with theoretical formulations developed and shaped by Western research.

Coping with COVID-19-mandated confinements, Ugarte Pérez et al. adapted two weight-loss interventions to telehealth: Mindfulness-Based Eating Awareness Training (MB-EAT) and Behavioral Weight Loss (BWL) counseling. To test their relative efficacy, these authors recruited Chilean participants through online social network announcements to conduct a randomized clinical trial (RCT). The authors found that both interventions produced comparable significant increases in eating mindfulness and interoceptive body awareness, as well as significant decreases in binge eating and body mass index (BMI). That is, Ugarte Pérez et al. replicated findings previously reported for Anglophone samples from economically developed countries. However, these authors also reported increases in anxiety and depression at the end of treatment, a finding that contradicts previous Western investigations (e.g., Salvo et al., 2022).

Using data from the Chilean Health Survey 2016–2017, Nazar et al. investigated discrepancies between respondents' BMI and their perceived weight status across four categories: underweight, normal weight, overweight, and with obesity. They examined the extent to which the size and direction of the discrepancies were cross-sectionally predictive of engaging in weight control practices. As previous research from various countries, these authors found that about half of their sample misperceived their BMI, and misperceptions were more likely to be underestimations than overestimations, particularly among those with BMIs indicative of overweight or obesity. Importantly, the self-perception of being overweight or having obesity was a stronger predictor of engaging in weight control practices than having a BMI within the overweight or obesity ranges.

Finally, Cai et al., developed and validated a picture library of high-calorie (starches, salty, fatty, and sugary drinks) Chinese foods (PLCF) for use in food craving research. These authors demonstrated that craving reactivity to the food images correlated with food-addiction scores, a relationship often found with Western samples (e.g., Delgado-Rodríguez et al., 2023). However, whereas Western researchers often report that sweets and fatty foods elicit the strongest cravings (e.g., Gearhardt and Schulte, 2021), Cai et al.found that pictures of salty foods and sugary drinks evoked the strongest cravings. Their PLCF can be accessed online through a link within their manuscript.

In conclusion, this collection of research articles that included non-Western samples revealed findings that were largely congruent with theoretical formulations and empirical findings previously obtained with Western, Anglo samples. However, each of the studies also revealed nuanced and unique findings that validate the importance of continuing to expand research on food addiction, eating disorders, and body image by including culturally and internationally diverse samples.

## Author contributions

AC-B: Conceptualization, Writing—original draft, Writing—review and editing. PL-C: Writing—review and editing. CU: Writing—review and editing. SM-D: Writing—review and editing.
